# Deconstructing Signaling Pathways in Cancer for Optimizing Cancer Combination Therapies

**DOI:** 10.3390/ijms18061258

**Published:** 2017-06-12

**Authors:** Ryuji Yamaguchi, Guy Perkins

**Affiliations:** 1 Department of Anesthesia, Kansai Medical University, Hirakata, Osaka 573-1010, Japan; 2 National Center for Microscopy and Imaging Research, School of Medicine, University of California San Diego, La Jolla, CA 92093, USA; perkins@ncmir.ucsd.edu

**Keywords:** combination therapy, lynchpin, signal transduction, apoptosis, VHL, PI3K, AKT

## Abstract

A single cancer cell left behind after surgery and/or chemotherapy could cause a recurrence of cancer. It is our belief that the failure of chemotherapies is the failure to induce apoptosis in all cancer cells. Given the extraordinary heterogeneity of cancer, it is very difficult to eliminate all cancer cells with a single agent targeting a particular gene product. Furthermore, combinations of any two or three agents exhibiting some proven efficacy on a particular cancer type have not fared better, often compounding adverse effects without evidence of expected synergistic effects. Thus, it is imperative that a way be found to select candidates that when combined, will (1) synergize, making the combination therapy greater than the sum of its parts, and (2) target all the cancer cells in a patient. In this article, we discuss our experience and relation to current evidence in the cancer treatment literature in which, by deconstructing signaling networks, we have identified a lynchpin that connects the growth signals present in cancer with mitochondria-dependent apoptotic pathways. By targeting this lynchpin, we have added a key component to a combination therapy that sensitizes cancer cells for apoptosis.

## 1. Introduction

Several studies showed that a single cancer cell grafted onto an animal genetically identical to a control animal caused cancer [1,2]. By extension, a scenario in which all cancer cells in a patient had not been removed by surgery or by chemotherapy, there would be a risk for cancer recurrence. Given the heterogeneity of cancer [3,4], this failure to remove all cancer cells is likely the reason why a single-agent therapy targeting a particular gene product has failed so many times [5]. Furthermore, a combination of any two or three agents exhibiting some proven efficacy on a particular cancer type often compounds adverse effects and furthermore does not necessarily improve the outcome [6,7]. What is needed is a rational system to combine chemotherapeutics.

In the past several years, our aim has been to assemble a combination of agents to treat von Hippel–Lindau (*VHL*)-deficient renal cancer. Since the VHL protein (pVHL) inhibits and its loss induces increased expression of the insulin-like growth factor 1 receptor (IGF1R) [8], IGF1R may seem like a good target for *VHL*-deficient renal cancer. However, a likely confounding effect is that epidermal growth factor receptor (EGFR), insulin receptors, and other receptor tyrosine kinases (RTKs) may also be expressed within the same tumor mass [9,10], activating many of the same signal transduction pathways. Thus a therapy based on an agent that targets only one of these receptors would likely leave many cancer cells unaffected.

There are more problems with a single agent therapy using a specific RTK inhibitor; for example, when H1650, a lung cancer cell line expressing the Gefitinib-sensitive EGFR-mutant, was treated with Gefitinib, it caused cells to exit the cell cycle, but it did not induce apoptosis [11]. In contrast, in another lung cancer cell line, H3255, expressing a different Gefitinib-sensitive EGFR mutant, Gefitinib induced apoptosis. In mice bearing H3255, treatment with Gefitinib caused tumor regression while with mice bearing H1650, Gefitinib only retarded tumor growth. Gefitinib has many adverse effects [12], prohibiting its use as a prophylactic agent, and thus making Gefitinib inappropriate for treating lung cancer in H1650-like cells. It is clear that in both cases, EGFR is driving cell proliferation in these cancer cells, but inhibiting it does not always induce apoptosis. Thus, in the pre-clinical phase we should aim for a combination that induces apoptosis in cultured cells and tumor regression in mouse models.

## 2. Inducing Apoptosis in Cancer Cells with Growth-Signal Inhibitors

Taking a step back, we ask, “How does the simple act of inhibiting EGFR induce apoptosis in some cancer cells?” If the growth signal is taken away, it seems more reasonable that cells should simply exit the cell cycle, as the Gefitinib-treated H1650 cells did. What is puzzling is that Gefitinib induced apoptosis in H3255 cells. In H3255 cells, even though extracellular signal–regulated kinase (ERK) activities were diminished by Gefitinib, they still persisted for more than 48 h, while AKT/protein kinase B activities were diminished almost completely immediately upon Gefitinib treatment. In contrast, in H1650 cells, AKT activities persisted for more than 48 h, while ERK activities were diminished immediately. One of the reasons for the differences is the absence of the phosphatase and tensin homolog (PTEN), a PtdIns(3,4,5)P3 phosphatase, in H1650 cells. In its absence, PtdIns(3,4,5)P3 accumulates, stimulating AKT [13]. Thus, AKT activity can persist for as long as 48 h in Gefitinib-treated *PTEN*-deficient H1650 cells [14]. Since AKT activity is known to interfere with apoptotic signals [15], it seems likely that AKT is protecting H1650 from entering apoptosis. But why were there persistent ERK activities in H3255 cells, and why did they die from the Gefitinib treatment?

One model explaining this phenomenon is that in H3255 cells, residual ERK activity or ERK activated by another RTK is driving the cell cycle in the absence of AKT. ERK drives the cell cycle machinery by transcriptionally activating many cell-cycle related genes, while activated AKT diverts resources from mitochondria for macromolecule and lipid synthesis [16,17] necessary for cell division. It also activates anti-apoptotic functions at the mitochondria [18], and it inactivates the cyclin-dependent kinase (CDK) inhibitor p27 [19]. In Gefitinib treated H3255, the balance between these two signals is disrupted in favor of proliferation, and the cell tries to proceed with cell division, but since cell division cannot take place in the absence of newly synthesized lipids and macromolecules, apoptosis is induced. Indeed, in most healthy and cancer cells, ERK and AKT activities are regulated in a highly cooperative manner [20]. Thus, according to this model, agents that block phosphoinositide 3-kinase (PI3K)-AKT, while leaving Ras-ERK unaffected, would be able to induce apoptosis. By extension, even cells with highly elevated ERK activity without concomitant AKT activity would be at risk. In support of this model, Shojaee and colleagues found that acute activation of oncogenes such as BCR-ABL and NRAS^G12D^ induce cell death in the vast majority of human pre-B cells [21]. In these cells, BCR-ABL (breakpoint cluster region–Abelson) and NRAS (neuroblastoma rat sarcoma) directly activate ERK, while the PI3K-AKT pathway that is normally activated by both RTK and Ras, is only activated by Ras. Some cells survived acute activation of BCR-ABL and NRAS^G12V^ by expressing high levels of negative regulators of ERK such as DUSP6, ETV5 and SPRY2 [21]. Presumably these negative regulators lowered ERK levels without affecting AKT levels, suggesting that in dying cells, AKT activities could not meet the demands for macromolecule and lipid syntheses necessary for rapid cell division. Furthermore, a small inhibitor of DUSP6 selectively induced cell death in patient-derived pre-B ALL (acute lymphoblastic leukemia) cells [21], suggesting that elevated levels of ERK without a concomitant elevation of AKT induces cell death.

Paralleling the above situation with the differential effects of the EGFR inhibitor Gefitinib on lung cancer cell lines are the differential sensitivities to the Her2 inhibitor Trastuzumab on BT474 and SKBR-3 breast cancer cell lines; Trastuzumab completely inhibits AKT but only partially inhibits mitogen-activated protein kinase (MAPK) in SKBR-3 cells, while it completely inhibits MAPK but only partially inhibits AKT in BT474 [22]. As expected, Trastuzumab induced apoptosis in SKBR-3 cells, while only arresting the cell cycle in BT474 cells [23]. In BT474 cells, however, it is not the loss of PTEN that supports AKT activity in the absence of upstream kinase activity, rather it is the constitutively active mutation in PINK (PTEN-induced putative kinase) 3CA [24].

## 3. Ras-ERK Pathways vs. PI3K-AKT Pathways

If the above model is correct, inhibitors of PI3K-AKT should be potent inducers of apoptosis in rapidly proliferating cancer cells [25], while inhibiting the Ras-ERK pathway is more likely to arrest cell growth [26], and it is less likely to induce apoptosis. In fact, most of the MAPK/ERK inhibitors had failed to show sufficient benefits in the clinical trials of non-small-cell lung, breast, colon and pancreatic cancers [27,28]. There are exceptions; ERK inhibitors induce apoptosis in some BRAF mutated melanoma cell lines in which ERK is activated independent of RTKs [29], and in the subset of osteosarcoma cell lines with constitutively activated ERK kinases [30]. The reason seems to be that MEK/ERK negatively regulates pro-apoptotic BIM (Bcl-2 Like Protein 11) expression through its phosphorylation [31] and through its translation [32]. Thus, in these cells, activated ERK prevents BIM from inducing apoptosis, and MEK/ERK inhibitors induce apoptosis by enhancing the expression of BIM [33]. In fact, restoring phospho-ERK activity allows melanoma cells to escape from BRAF inhibitor therapy [34]. Thus, these cancer cells come to rely heavily on the activities of two proteins, ERK1 and ERK2, for their survival. However, since ERK inhibitors induce apoptosis only in a small number of cells in a tumor, they do not show significant activities in clinical trials of soft-tissue-sarcoma and uveal melanoma [35,36].

Even though there are examples in which PI3K inhibitors induced cell cycle arrest [37], many believe that PI3K-AKT pathway inhibitors are more likely to induce apoptosis in cancer cells. This is because most of the PI3K inhibitors induce apoptosis in healthy proliferating cells. For example, LY294002 is a reversible inhibitor of PI3K, yet injecting it at effective doses into mice would be lethal [38]. Thus, while they kill cancer cells, these inhibitors would also kill healthy, proliferating cells. To circumvent the adverse effects of PI3K inhibitors, common strategies have been to develop isoform-specific inhibitors of PI3K-AKT pathways with increasing specificities [39]. In clinical trials, most of these inhibitors have been well tolerated, but they only showed modest single-agent clinical activities [7].

## 4. Inducing Apoptosis in *VHL*-Deficient Cancer Cells

The most well-known role for pVHL is in the ubiquitination and degradation of hypoxia-inducible factors under normoxic conditions. In *VHL*-deficient cancer cells, hypoxia-inducible factors are constitutively activated [40], targeting many hundreds of genes for transcriptional activation. Most of these genes facilitate metabolic adaptation to hypoxia. Furthermore, the expression of these hypoxia-inducible proteins is known to make cancer cells resistant to chemotherapeutic agents [41]. However, there are exceptions; mitochondria-targeting agents such as the B-cell lymphoma 2 (Bcl-2) antagonist ABT (Abbott)-263 (ABT), alone or together with 2-deoxyglucose (2DG), were reported to induce apoptosis under hypoxic conditions just as efficiently as under normoxic conditions in breast cancer and renal cancer cells [42,43,44] (in fact, ABT can induce apoptosis in quiescent cells as well [45,46]). Furthermore, since *VHL*-deficient cells are more glycolytic, increasing glucose uptake, they also have higher uptake of 2DG. Since the 2DG-ABT combination induces apoptosis by directly activating pro-apoptotic Bak/Bax (Bcl-2 homologous antagonist-killer/Bcl-2 associated x protein) at mitochondria, thus circumventing the many steps usually required for apoptosis induction, we expected the 2DG-ABT combination to induce apoptosis very quickly and very efficiently. Normally Bak/Bax is bound to Mcl-1 and Bcl-xL, inhibiting its activation. Because ABT binds to the Bcl-2 family of proteins with high affinity with the exception of Mcl-1, treating cells with ABT causes the dissociation of Bcl-xL from Bak/Bax. On the other hand, 2DG-induced stress at mitochondria causes the dissociation of Mcl-1 from Bak/Bax. The precise molecular mechanism for this is unknown. The dual treatment of cancer cells releases Bak/Bax from its inhibitory association with Mcl-1 and Bcl-xL. Once Bak/Bax is activated, it forms a pore on the outer membrane of mitochondria, releasing cytochrome *c* into the cytosol. The released cytochrome *c* assembles apoptosome complexes, large caspase complexes that lyse many cellular proteins. A single treatment with 2DG-ABT combination induced apoptosis in over 94% of MCF-7 breast cancer, PPC-1 prostate cancer cells and in many other cell lines [47]. However, apoptotic rates were well below 50% in *VHL*-deficient renal cancer cells lines such as RCC4 and UOK121 [44,48]. In these *VHL*-deficient cell lines, apoptotic rates did not change greatly, whether the experiments were performed under hypoxic (1% O_2_) or normoxic (21% O_2_) conditions. Forced expression of functional pVHL in *VHL*-deficient cells comparable to *VHL* expressed in other renal cancer cells [44], on the other hand, raised their sensitivity to 2DG-ABT, even though the apoptotic rates remained the same between hypoxic and normoxic cells. Taken together, these data suggest pVHL may play a role in sensitizing cancer cells for mitochondria-targeting drugs independent of the oxygen concentration.

## 5. Deconstructing Signaling Pathways in *VHL*-Deficient Cancer Cells

pVHL is also known to play a role in a few cellular processes independent of oxygen concentration, such as the assembly and regulation of the extracellular matrix, the stabilization of microtubules, transcriptional regulation, and others (reviewed by Gossage et al. 2015 [49]). In particular, IGF1R is upregulated in the absence of functional pVHL independent of the oxygen concentration [8,44]. Depleting IGF1R by siRNA sensitized *VHL*-deficient cells for 2DG-ABT treatment, suggesting that *VHL*-deficient cancer cells are resistant to 2DG-ABT because they express IGF1R.

IGF1R, EGFR and many other RTK share common response pathways; they all activate both the Ras-ERK proliferation pathway and the PI3K-AKT pro-survival pathway. Activated Ras activates Raf, which in turn activates ERK signals [50]. Activated Ras also directly activates PI3K [51], generating cross talk between these two pathways in the early stages of signal transduction. However, when these signals reach PI3K and ERK, the two pathways can be clearly distinguished. By using specific inhibitors of ERK and PI3K, we identified which of the two pathways interferes with 2DG-ABT-induced apoptosis. We identified the PI3K-AKT pathway as the effecting pathway.

**Figure 1:**
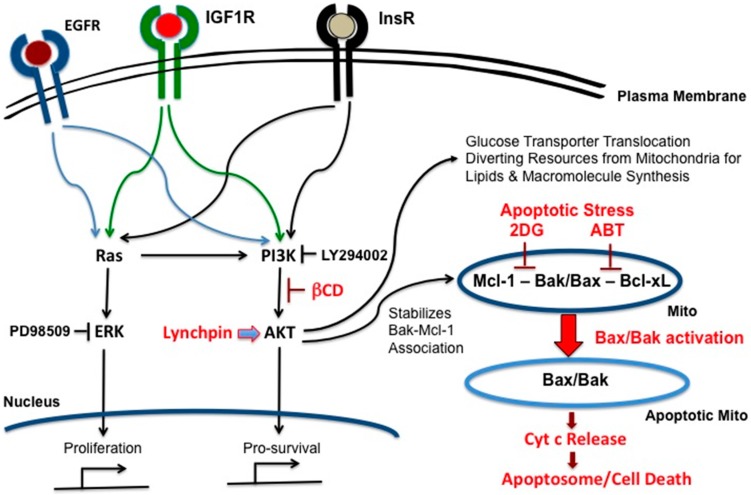
Phosphoinositide 3-kinase (PI3K)-AKT is the lynchpin that connects growth signals present in cancer cells with mitochondria-dependent apoptotic pathways centered on Bax and Bak. The epidermal growth factor receptor (EGFR), insulin-like growth factor 1 receptor (IGF1R) and InsR receptors on the plasma membrane modulate both Ras and PI3K-AKT signaling pathways, thus activating the proliferation and pro-survival responses, respectively. AKT stabilizes the Mcl-1/Bak association, and thus impedes Bax/Bak induced apoptosis; βCD: β-cyclodextrin.

Treatment of *VHL*-deficient cancer cells with a specific inhibitor of PI3K sensitizes cells for 2DG-ABT, but not by using an ERK inhibitor under normoxic conditions. Thus, *VHL*-deficient cells gain resistance to 2DG-ABT through the raised expression of IGF1R and subsequent activation of PI3K-AKT, and not by activating hypoxia-inducible factors which they cannot activate [44]. But more importantly, we found the lynchpin between the 2DG-ABT apoptotic pathway and growth signals in *VHL*-deficient cells was PI3K-AKT (Figure 1).

## 6. Targeting Mcl-1

In order to determine the targets of AKT, we depleted AKT with siRNA. In the absence of AKT, ABT alone could induce apoptosis, and it did not need 2DG. In these cells, the AKT that normally up-regulates Mcl-1 expression is absent, and Mcl-1 protein levels decline. Bak/Bax normally sequestered in its inhibitory association with Mcl-1 and the Bcl-2 family of proteins, can now be activated by ABT alone, inducing apoptosis. Thus, the combination of AKT knockdown and ABT can induce apoptosis in almost any cell, including healthy proliferating cells, as well as quiescent cells. This is a very toxic effect.

In contrast, application of LY294002, a specific inhibitor of PI3K, inactivated AKT almost immediately, but did not lower Mcl-1 protein levels (The inhibitory effect of LY294002 lasts only 8 h in cultured cells [52]), and yet it still sensitized cells to 2DG-ABT, but not to ABT alone. In these cells, Mcl-1 dissociated from pro-apoptotic Bak only when cells were treated with both 2DG and LY294002. Thus, phospho-AKT directly interferes with 2DG-induced changes at mitochondria, stabilizing the association between anti-apoptotic Mcl-1 and pro-apoptotic Bak [44]. The effect of the combination of LY294002 and 2DG-ABT are restricted to highly glycolytic cells such as rapidly growing cancer cells, which is exactly what is desired. Unfortunately, LY294002 has many adverse effects, and is not appropriate for clinical use.

## 7. Cholesterol-Targeting Drugs Attenuate PI3K-AKT Signals

**Figure 2:**
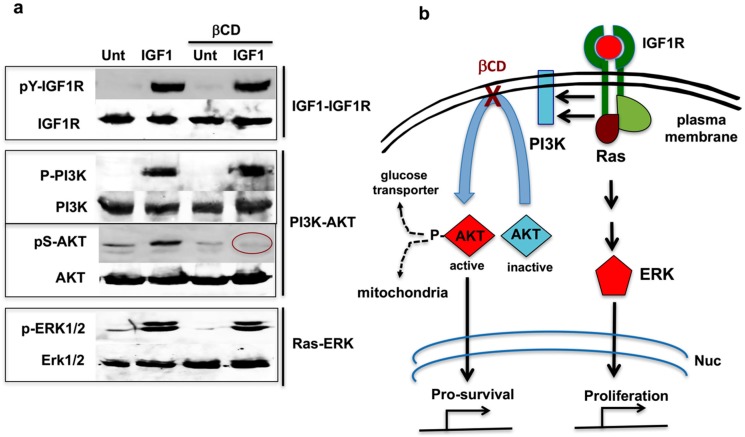
β-Cyclodextrin (βCD) interrupts PI3K-AKT signal transduction. (**a**) Western blots showing that βCD interrupts signal transduction between PI3K and AKT, while the Ras-ERK pathway remains intact; and (**b**) Schematic indicating that βCD interferes with RTK–PI3K–AKT pro-survival signals while leaving RTK–Ras–ERK proliferation signals intact. Nuc (nucleus). Method for the results shown in Figure 2a. HeLa cells were serum-starved for 60 min in high glucose DMEM (4.5 g/L). In the last 30 min of this incubation, 10 mM methyl-β-cyclodextrin—indicated by βCD (MBCD) was added to the cells as indicated. Then 20 ng/mL of IGF1 was added to the samples, and incubation continued for another 20 min. Cells were harvested and analyzed by Western blotting with the indicated antibodies. For more examples and details, see Yamaguchi et al. [48].

It has been proposed that cholesterol-containing lipid rafts are central for efficient signal transduction from the plasma membrane to the cytosol [53]. However, we were surprised to find that its effects were limited to certain signals such as signaling from PI3K to AKT [48]. Cholesterol-targeting drugs such as β-cyclodextrin (βCD) and its derivatives, methyl-β-cyclodextrin (MBCD) and 2-hydoxoypropyl-β-cyclodextrin (HPBCD), can sequester cholesterol in their hydrophobic cores and temporally deplete cholesterol from the plasma membrane, but not phospholipids [54]. In the absence of cholesterol at the plasma membrane, AKT could not be activated by PI3K. Thus, IGF1R can activate the Ras-ERK proliferation signal, while the PI3K-AKT pro-survival signal is interrupted between the PI3K and AKT step (Figure 2).

How treatments with βCD hinder signal transduction between PI3K and AKT is not fully understood. Since myristoylated AKT was activated by PI3K in the presence of βCD, it seems likely that in the absence of cholesterol, AKT could not be recruited to the plasma membrane (unpublished observation). However, it is possible that the altered cholesterol trafficking in βCD-treated cells inactivates mTORC2, and that in turn blocks AKT activation [55,56,57]. More research is needed to resolve the precise molecular mechanism of how βCD disrupts the signal transduction between PI3K and AKT.

We also found that a pharmacological dose of βCD (50 mg/kg) can block the signal from IGF1R to AKT, attenuating IGF1-induced hypoglycemia in the body, providing an assay to determine the duration of the inhibitory effect in the animal body. When βCD was injected in mice, it attenuated IGF1-induced hypoglycemia [48] for approximately 4 h and otherwise caused little toxicity, suggesting that the apoptotic pathway must be activated within a short period. βCD stimulates glucose and therefore 2DG uptake [58]. However, it also reduces 2DG uptake, by attenuating insulin or IGF1-(both present in the serum) stimulated glucose uptake. Lastly, βCD and its derivatives cannot cross the blood-brain barrier, thus limiting their effect to tissues outside the brain. Taking these issues into consideration, we developed a protocol for combining three agents into a therapeutic protocol.

## 8. The Combination Therapy

We found that the following protocol was efficacious as a combination therapy: add 2DG first, 90 min later, add βCD, and 30 min later, add ABT. When cultured *VHL*-deficient cells were treated with this protocol, cytochrome *c* was released and over 90% of cells were dead within 16 h. This was true even when we washed the cells free of all these reagents within four hours from the start of the treatment to mimic what cancer cells in the body would experience [44,48]. When mice bearing UOK121 cells were treated with a triple combination (using HPBCD in place of βCD), we observed tumor regression [48]. Unfortunately, the triple combination caused a cachexia-like condition in treated mice. None of the other treatments we tried caused the cachexia-like condition.

All three agents were applied at pharmacological concentrations. 2DG is used at 50 mg/kg, three times a week for three weeks. Known adverse effects of 2DG occur at higher concentrations and/or when administered for longer periods. For example, (1) a one-time administration of 2DG over 500 mg/kg caused acute hypoglycemia-like conditions in humans within minutes [59], and (2) feeding rats with 2DG at approximately 200 mg/kg/day over 50 weeks induced cardiac vacuolization and increased mortality [60]. On the other hand, feeding rats 2DG below 100 mg/kg/day resulted in the mortality rate being almost identical to the control population [60].

Even at pharmacological doses, ABT is known to cause lymphopenia and thrombopenia [61]. Combined with 2DG, however, we observed little ill effects other than lowered blood counts in immunocompromized mice [47], and they survived the regimen well without loss of body weight. 

Because βCD and its derivatives can sequester hydrophobic molecules in their core, they have been used as a delivery vehicle for pharmacological agents [62]. Their safety has been extensively tested in clinical studies [63]. Furthermore, HPBCD is in clinical trials for treating Niemann-Pick Type c disease. Even though the pharmacological dose of HPBCD is 40 mg/kg, healthy volunteers have taken 470 mg/kg/day HPBCD for four days, and up to 3 g in a single dose with no apparent ill effect [63]. Because all three agents affect metabolism of cancer cells—2DG by reducing glucose metabolism, βCD by temporally attenuating AKT and hexokinase activities at mitochondria, and ABT by affecting Bcl-w involved in mitochondria fusion [47]—it is possible that all three agents together perturb cellular metabolism in a way that causes the observed cachexia-like condition. However, we have not yet determined precisely the cause of this complication.

## 9. Future of the Triple Combination

Each of the three agents we used (HPBCD, 2DG, ABT), have all been tested in clinical trials. At the established pharmacological doses, only ABT has known side effects: lymphopenia and thrombopenia [64]. Injected into the body, 2DG accumulates in cells with elevated glucose uptake such as brain cells, cells in inflamed tissues, muscle cells under heavy exertion, and cancer cells. Because HPBCD and ABT cannot cross the blood-brain barrier, under the proper treatment condition, only highly glycolytic cancer cells outside the brain would be exposed to all three agents, thus limiting the adverse effects of the combination therapy. As a single agent, none of them were effective in treating *VHL*-deficient renal cancer in mouse models. Only the triple combination induced tumor regression, providing a proof of principle for our strategy. It remains to be seen if the cachexia-like condition is an unavoidable consequence of the triple combination, or if it can be avoided by using another Bcl-2 antagonist with fewer side effects such as ABT-199 [65,66] and possibly others [67], by using a different variant of βCD [62], by blocking myostatin, which is an endogenous negative regulator of muscle growth [68,69], or perhaps using the drugs enobosarm or anamorelin to inhibit cachexia [70].

## 10. Two Switches for Inducing Apoptosis

Unwanted cells can be eliminated by CD8+ cytoxic T cells using a Granzyme B (GzmB) injection and perforin. Normally, GzmB cleaves a pro-apoptotic member of the Bcl-2 family, BID (BH3 interacting-domain death agonist), for activation. However, GzmB-targeted cells need not have BID or Bak/Bax, and need not release cytochrome *c* from mitochondria to die from this treatment. Thus, even though most of the targeted cells die of mitochondria-dependent apoptosis, they can also in fact die without the activation of the mitochondria-dependent apoptotic machinery. Therefore, the focus of immunotherapy research has been on how cytoxic T cells become activated, and not on how apoptosis is induced in the targeted cell. In contrast, the aim of chemotherapies is to induce apoptosis in all cancer cells, and if a chemotherapy does not involve the death receptor ligands, the eventual goal is to activate Bak/Bax at mitochondria. Thus, how Bak/Bax is activated is of paramount importance.

**Figure 3:**
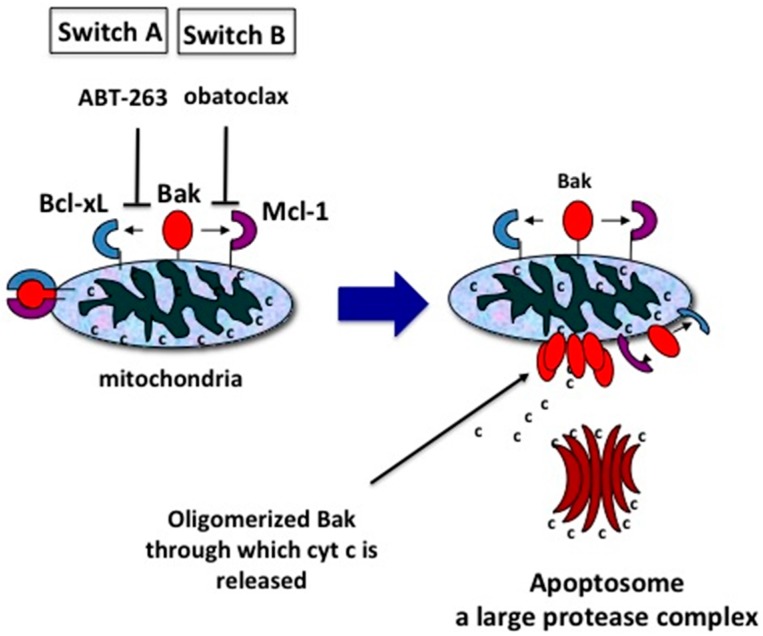
Two switches for inducing apoptosis. Switch A dissociates Bak-Bcl-xL. Switch B dissociates Bak-Mcl-1. These associations can be broken by the molecular inhibitors, ABT-263 and obatoclax, respectively. For simplicity, only Bak and Bcl-xL are depicted instead of Bak/Bax and Bcl-2, Bcl-w, Bcl-xL respectively.

Bak/Bax is normally sequestered by its inhibitory association with Mcl-1 and the rest of the Bcl-2 family (Bcl-2, Bcl-xL and Bcl-w). BH3-only proteins such as truncated BID or BIM can bind to all Bcl-2 family proteins, freeing Bak/Bax from all its inhibitory associations, and triggering apoptosis. Small molecule inhibitors such as ABT can bind to Bcl-2, Bcl-xL and Bcl-w, dissociating them from Bak/Bax [71], while obatoclax and A-1210477 can bind to Mcl-1, dissociating it from Bak/Bax [71,72], and application of both induce apoptosis in almost any cell, including healthy quiescent cells [46]. Thus, from our perspective, there are two switches for inducing apoptosis, the Bcl-2 switch (Switch A) and the Mcl-1 switch (Switch B), and when both switches are on, apoptosis is induced in almost any cell in the body, including healthy cells, as well as extensively mutated cancer cells such as PTEN-deleted p53 mutated highly chemoresistant prostate cancer cells [47] (Figure 3).

**Figure 4:**
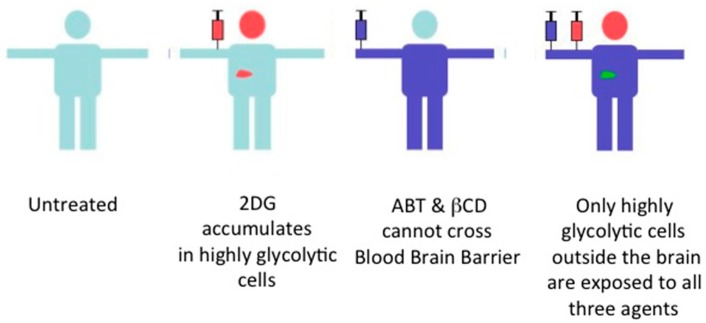
The combination therapy has a built-in limit to the type of cells affected. In this scheme, only highly glycolytic cancer cells outside the brain are exposed to 2DG, βCD and ABT.

Of the two switches, Mcl-1 is the more interesting case. This is because the association of Mcl-1 to Bak/Bax is regulated at multiple levels. Mcl-1’s expression level is regulated by transcriptional [73], post-transcriptional [43] and post-translational processes [74,75]. Furthermore, its association with Bak is regulated by AKT [44,76], and is lost when certain cell types are treated with 2DG [47] or when cell attachment is disrupted in adhesive cells (our unpublished observation). Freed from Bak/Bax, Mcl-1 associates with Caveolin-1, which seems to stabilize Mcl-1 during cell-detachment [77,78,79]. Furthermore, in HL-60, human promyelocytic leukemia cells, Mcl-1 does not associate with Bak/Bax, but is maintained in the cytosol [80]. Thus, there are several ways in which Mcl-1 association with Bak/Bax can be manipulated in cells with elevated glucose uptake [44,47,48]. With more detailed knowledge, we may find many more ways to turn on Switch B. On the other hand, Switch A can always be turned on with a Bcl-2 antagonist that cannot cross the blood-brain barrier such as ABT. Thus, during treatment the only cells in the body with both switches on are highly glycolytic cells outside the brain (Figure 4).

Once Switch B is turned on in highly glycolytic cells, they are sensitized not only for mitochondria-dependent apoptosis, but also for the Tnf-α-Related Apotosis Inducing Ligand (TRAIL) [48]. This is because TRAIL-activated caspase cascades can cleave BID. Even though the amount of activated BID is low, because mitochondria are sensitized by Mcl-1 loss, cytochrome *c* can still be released, which in turn activates more capsases, thus creating a signal amplification loop, inducing apoptosis with a small amount of TRAIL. Because TRAIL antibodies cannot cross the blood-brain barrier, the combination should not affect brain cells, adding an extra safety feature to the combination therapy. In fact, the combination of 2DG-βCD-TRAIL induced apoptosis in difficult-to-treat pancreatic cancer cells [48].

## 11. Summary

Trying to induce apoptosis in cancer cells by manipulating growth signals and/or cell cycle checkpoints is difficult because there are so many signaling steps between the elements directly affected by chemotherapeutics and the final steps in apoptosis, and defects at any step can compromise apoptosis induction. On the other hand, manipulating Bak/Bax inhibitors can easily induce apoptosis. By carefully dissecting molecular machineries blocking Bak/Bax activation, we believe that it is possible to find a combination that induces apoptosis efficiently in all cancer cells across varied genetic backgrounds.
